# Supplementary material for: Intrahippocampal Inoculation of Aβ1–42 Peptide in Rat as a Model of Alzheimer’s Disease Identified MicroRNA-146a-5p as Blood Marker with Anti-Inflammatory Function in Astrocyte Cells
Source: Cells. 2023 Feb 22;12(5):694. doi: 10.3390/cells12050694 (PMC10000752; doi:10.3390/cells12050694)
Supplement: Supplementary file 1 [file cells-12-00694-s001.zip › cells-2142170-supplementary.pdf]

Supplementary data

Table 1. Sequence of microRNA primers

mature microRNA	Sequence	Sanger Accesion	Catalog number
rno-miR-9a-5p	UCUUUGGUUAUCUAGCUGUAUGA	MIMAT0000781	MS00013951
rno-miR-146a-5p	UGAGAACUGAAUUCCAUGGGUU	MIMAT0000852	MS00000441
rno-miR-29a-3p	UAGCACCAUCUGAAAUCGGUUA	MIMAT0000802	MS00033397
rno-miR-29c-3p	UAGCACCAUUUGAAAUCGGUUA	MIMAT0000803	MS00000175
cel-miR-39-3p	UCACCGGGUGUAAAUCAGCUUG	MIMAT0000010	MS00019789
cel-miR-238-3p	UUUGUACUCCGAUGCCAUUCAGA	MIMAT0000293	MS00019439
cel-miR-248	AUACACGUGCACGGAUAACGCUCA	MIMAT0000304	MS00019516

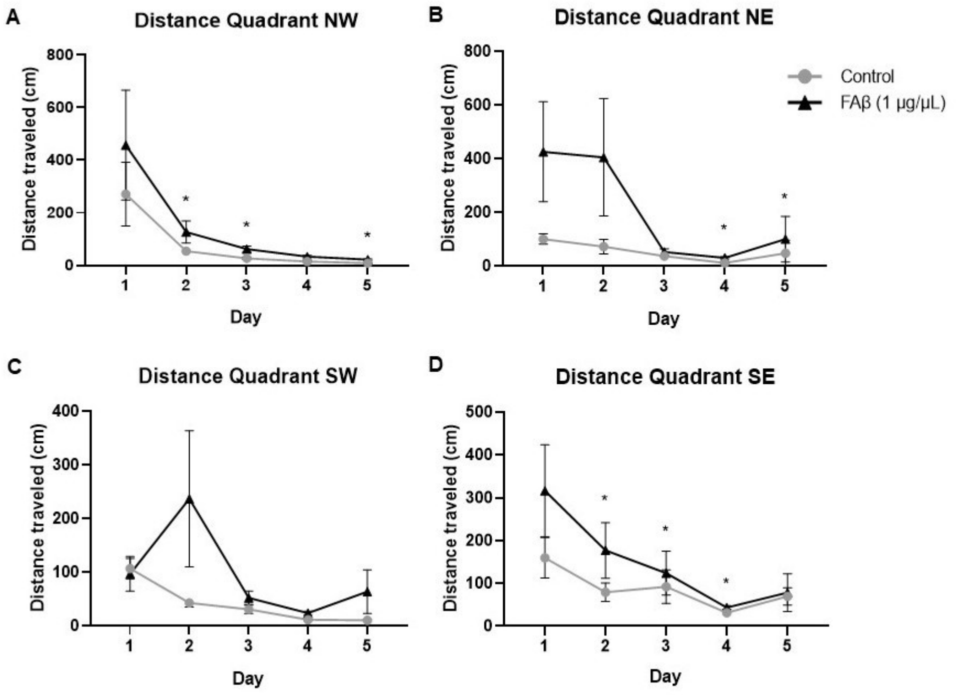

Figure S1. Effects of $FA\beta_{1-42}$ injection on memory and learning evaluated by the Morris Water Maze test. The distance performed by rats (n = 10) injected with $Fa\beta_{1-42}$ peptide solutions (1 µg/µL) was evaluated, at day 14 post-injection. Distance (A) in the quadrant NW (B) in the quadrant NE, (C) in the quadrant SW (D), in the target quadrant SE. Control

group of rats (n=10) was injected with PBS solution. Data are represented as mean ± SEM. Statistical comparisons were performed using Kruskal-Wallis. *$p \leq 0.05$.

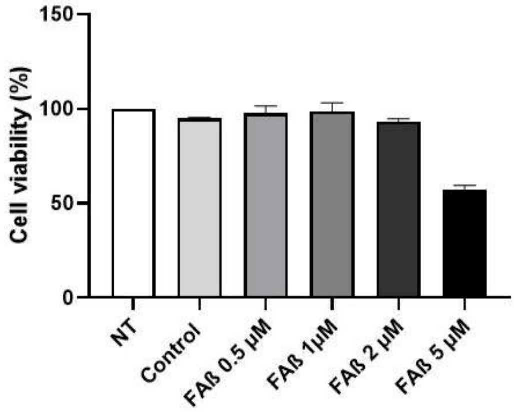

Figure S2. Incubation of primary astrocytes cell culture with FA□$_{1-42}$ peptides solutions at indicated concentrations did not lead to any cellular toxicity. Alamar Blue cell viability assay of primary Astrocyte cell culture treated with FA□$_{1-42}$ peptide solutions. The fluorescence value from the untreated cells (NT) was set up as the arbitral value of 100 %.
